# Survival Impact of Current-Smoking-Related COPD or COPD with Acute Exacerbation on Bladder Preservation through Concurrent Chemoradiotherapy for Muscle-Invasive Bladder Urothelial Carcinoma

**DOI:** 10.3390/jpm11100958

**Published:** 2021-09-26

**Authors:** Jiaqiang Zhang, Shyh-Chyi Chang, Ming-Feng Chiang, Kuo-Chin Chiu, Szu-Yuan Wu

**Affiliations:** 1 Department of Anesthesiology and Perioperative Medicine, People’s Hospital of Zhengzhou University, Henan Provincial People’s Hospital, Zhengzhou 450052, China; jiaqiang197628@163.com; 2 Department of Surgery, Division of Urology, Lotung Poh-Ai Hospital, Yilan 256, Taiwan; mork2747@gmail.com; 3 Faculty of Medicine, National Yang-Ming University School of Medicine, Taipei 11221, Taiwan; 4 Division of Gastroenterology and Hepatology, Department of Internal Medicine, Lo-Hsu Medical Foundation, Lotung Poh-Ai Hospital, Yilan 256, Taiwan; chiangmingf@gmail.com; 5 Division of Chest, Department of Internal Medicine, Lo-Hsu Medical Foundation, Lotung Poh-Ai Hospital, Yilan 256, Taiwan; chiukc1@yahoo.com.tw; 6 Department of Food Nutrition and Health Biotechnology, College of Medical and Health Science, Asia University, Taichung 413, Taiwan; 7 Big Data Center, Lo-Hsu Medical Foundation, Lotung Poh-Ai Hospital, Yilan 256, Taiwan; 8 Division of Radiation Oncology, Lo-Hsu Medical Foundation, Lotung Poh-Ai Hospital, Yilan 256, Taiwan; 9 Department of Healthcare Administration, College of Medical and Health Science, Asia University, Taichung 413, Taiwan; 10 Cancer Center, Lo-Hsu Medical Foundation, Lotung Poh-Ai Hospital, Yilan 256, Taiwan; 11 Graduate Institute of Business Administration, Fu Jen Catholic University, Taipei 242062, Taiwan; 12 Centers for Regional Anesthesia and Pain Medicine, Taipei Municipal Wan Fang Hospital, Taipei Medical University, Taipei 110, Taiwan

**Keywords:** muscle-invasive bladder urothelial carcinoma, COPD, COPDAE, cigarette smoking, survival

## Abstract

**PURPOSE:** The survival effect of smoking-related chronic obstructive pulmonary disease (COPD) and COPD with acute exacerbation (COPDAE) on patients with muscle-invasive bladder urothelial carcinoma (MIBUC) receiving concurrent chemoradiotherapy (CCRT) for bladder preservation is unclear. **METHODS:** We recruited patients with MIBUC, clinical stages IIA–IVB, who had received maximal transurethral resection of bladder tumor (TURBT) followed by CCRT from the Taiwan Cancer Registry Database. The Cox proportional hazards model was used to analyze all-cause mortality. We categorized the patients into two groups by using propensity score matching based on the preexisting COPD status (within 1 year before CCRT) to compare overall survival outcomes: Group 1 (never smokers without COPD) and Group 2 (current smokers with COPD). **RESULTS:** In multivariate Cox regression analyses, the adjusted hazard ratio (aHR; 95% confidence interval (CI)) of all-cause mortality in Group 2 compared with Group 1 was 1.89 (1.12–3.18), *p* = 0.017. The aHRs (95% CIs) of all-cause mortality for ≥1 and ≥2 hospitalizations for COPDAE within 1 year before CCRT for bladder preservation were 3.26 (1.95–5.46) and 6.33 (3.55–11.281) compared with non-COPDAE patients with MIBUC undergoing CCRT for bladder preservation. **CONCLUSIONS:** Among patients with MIBUC undergoing TURBT followed by CCRT for bladder preservation, current smokers with smoking-related COPD had worse survival outcomes than did nonsmokers without COPD. **CONDENSED ABSTRACT:** This was the first study to estimate the survival impact of smoking-related chronic obstructive pulmonary disease (COPD) on patients with muscle-invasive bladder urothelial carcinoma (MIBUC) receiving maximal transurethral resection of bladder tumor (TURBT) followed by concurrent chemoradiotherapy (CCRT) for bladder preservation. Smoking-related COPD was a significant independent risk factor for all-cause mortality in patients with clinical stages IIA–IVB receiving TURBT followed by CCRT. Hospitalization frequency for COPD with at least one acute exacerbation within 1 year before CCRT was highly associated with high mortality for patients with MIBUC receiving CCRT for bladder preservation. Not only all-cause death but also bladder cancer death and COPD death were significantly higher in the current-smoking COPD group than in the never-smoking non-COPD group.

## 1. Introduction

Bladder cancer is the most common malignancy of the urinary system, with approximately 84,000 new cases and 17,000 deaths in the United States annually [1]. Worldwide, bladder cancer accounts for approximately 600,000 new cases and >200,000 deaths per year [2]. In developed areas, such as North America, Western Europe, and Taiwan, bladder cancers are predominantly urothelial [1,3]. Moreover, in Taiwan, bladder cancer is the most common urinary malignancy [3], with approximately 2200 new cases and 1000 deaths annually [3]. Nearly 70% of patients with bladder cancer are diagnosed at an early stage [1,3], and the remaining develop muscle-invasive bladder urothelial carcinoma (MIBUC), in the muscularis propria (T2), perivesical tissue (T3), or adjacent pelvic structures (T4) [1,3].

Radical cystectomy with neoadjuvant cisplatin-based chemotherapy is the standard approach for the treatment of muscle-invasive urothelial bladder cancer [4]. For patients who are unsuitable for radical cystectomy or who desire preservation of their native bladder, trimodality therapy consisting of maximal transurethral resection of bladder tumor (TURBT) followed by concurrent chemoradiotherapy (CCRT) (trimodality bladder preservation treatment (TMT)) is an appropriate alternative [5]. The National Comprehensive Cancer Network (NCCN) guidelines recommend bladder preservation with CCRT (category 1 evidence) for MIBUC [6]. In the extended follow-up, CCRT for bladder preservation in MIBUC was associated with improved disease-specific survival and decreased rates of salvage radical cystectomy [7,8].

Smoking status is associated with decreased response rates to neoadjuvant chemotherapy of cisplatin-based regimens and increased overall and cancer-specific mortality as well as bladder cancer recurrence after radical cystectomy [9,10]. In addition, tobacco smoking is overwhelmingly the most important risk factor for chronic obstructive pulmonary disease (COPD) [11,12,13,14,15,16], and current smoking is common among patients with COPD with acute exacerbation (COPDAE) [17]. However, no study has shown the survival impact of smoking-related comorbidities, such as COPD and severe COPD (hospitalization for COPDAE), on patients with MIBUC receiving CCRT for bladder preservation. The valuable information of current-smoking-related COPD severity might be a prognostic factor for survival in patients with bladder cancer who receive CCRT for bladder preservation. Moreover, the prevention of COPD progression to COPDAE might be crucial for increasing overall survival (OS) in patients with MIBUC receiving definitive CCRT for bladder preservation if the severity of smoking-related COPD could affect OS in these patients. Therefore, we conducted a head-to-head propensity score matching (PSM) study to estimate the survival impact of current-smoking-related COPD and never-smoking patients without COPD with MIBUC receiving CCRT for bladder preservation.

## 2. Patients and Methods

### 2.1. Study Population

For this cohort study, we enrolled patients with a diagnosis of MIBUC between 1 January 2009, and 31 December 2018, from the Taiwan Cancer Registry Database (TCRD). The index date was the date of CCRT, and the follow-up duration was from the index date to 31 December 2019. The TCRD contains detailed cancer-related information of patients, including the clinical stage, cigarette smoking habit, surgical techniques, treatment modalities, pathologic data, irradiation doses, chemotherapy regimen and dosage, and differentiation grade [18,19,20,21,22]. The study protocols were reviewed and approved by the Institutional Review Board of Tzu-Chi Medical Foundation (IRB109-015-B).

### 2.2. Inclusion and Exclusion Criteria

The diagnoses of the enrolled patients were confirmed after reviewing their pathological data, and patients with newly diagnosed MIBUC were confirmed to have no other cancers or distant metastases. Bladder-preserving therapy in our study included trimodality therapy consisting of maximal TURBT followed by CCRT for patients with muscle-invasive bladder who were medically unfit for radical cystectomy or who wished to preserve their native bladder. In our study, cisplatin-based chemotherapy regimens were administered concurrently with radiotherapy. Patients were included if they had received an MIBUC diagnosis and TURBT followed by CCRT, were ≥20 years old, and had clinical T2a–T4 without metastasis according to the American Joint Committee on Cancer (AJCC, 8th edition) criteria. Clinical lymph node-positive patients were included for bladder preservation. In our study, the goal of TURBT was to maximally resect all visible tumors safely. Patients were excluded if they had a history of other cancers before the index date, unknown clinical stage, missing sex data, missing smoking records, unclear differentiation of tumor grade, or nonurothelial carcinoma. Patients who received an radiotherapy dose of <60 Gy were excluded because it is not the standard radiotherapy dose for bladder preservation according to the NCCN guidelines [6]. We categorized the enrolled patients into two groups based on their current smoking and COPD status to compare all-cause mortality: Group 1 (never smokers without COPD) and Group 2 (current smokers with smoking-related COPD). Furthermore, we estimated the survival outcome associated with the severity of smoking-related COPD (hospitalization frequency for COPDAE with 0, ≥1, and ≥2 hospitalizations within 1 year before the index date) and with patients with clinical stage IIA–IVB MIBUC undergoing CCRT for bladder preservation. Comorbidity incidence was scored using the Charlson comorbidity index (CCI) [23,24]. Diabetes, hyperlipidemia, hypertension, acute myocardial infarction (AMI), cardiovascular diseases, ischemic stroke, and kidney or bladder stones were excluded from the CCI scores to prevent repetitive adjustment in the multivariate analysis. Only comorbidities or COPD observed within 12 months before the index date were included based on the *International Classification of Diseases, 10th Revision, Clinical Modification* (*ICD-10-CM*) codes issued at the first admission or more than twice at outpatient department visits.

### 2.3. PSM and Covariates

To reduce the effects of potential confounders in the all-cause mortality between Groups 1 and 2, we performed 2:1 PSM with a caliper of 0.2 standard deviation for the following variables: age, sex, diabetes, hyperlipidemia, hypertension, AMI, cardiovascular diseases, ischemic stroke, kidney/bladder stones, CCI scores, AJCC clinical tumor stage, AJCC clinical nodal stage, surgical consolidation after CCRT, bladder preservation rate, cisplatin-based regimen dosage, and radiotherapy dosage. A Cox proportional hazards model was used to regress all-cause mortality of different COPD statuses, with a robust sandwich estimator used to account for clustering within matched sets [25]. Multivariate Cox regression analyses were performed to calculate hazard ratios to determine whether the COPD status and hospitalization frequency for COPDAE within 1 year before the index date are potential independent predictors of all-cause mortality. Age, sex, diabetes, hyperlipidemia, hypertension, AMI, cardiovascular diseases, ischemic stroke, kidney/bladder stones, CCI scores, AJCC clinical tumor stage, AJCC clinical nodal stage, surgical consolidation after CCRT, bladder preservation rate, cisplatin-based regimen dosage, and radiotherapy dosage might be prognostic factors of all-cause death for patients with MIBUC. Furthermore, although PSM was performed (Table 1), they might be the independent potential prognostic factors of all-cause death with residual imbalance [26,27]. We also supplied the data on the cohort before matching (Supplemental Table S1) for its internal validity. Cox regression was performed for these covariates (Table 2). Potential predictors were controlled for through PSM (Table 1), and all-cause mortality was the primary endpoint in both groups. COPD death and bladder cancer death estimations according to the Cause of Death database are presented in Table 1. With a well-matched PSM design, the real-world data can show the survival impact of COPD and COPDAE on all-cause death, COPD death, and bladder cancer death for patients with MIBUC receiving TURBT followed by CCRT for bladder preservation. We also supply the characteristics of non-COPDAE vs. COPDAE in the COPD group as supplemental Table S2 and multivariable analysis of the non-COPDAE and COPDAE in the COPD group as supplemental Table S3 to clarify the effect of COPDAE.

### 2.4. Statistics

After adjustment for confounders, all analyses were performed using SAS version 9.3 (SAS Institute, Cary, NC, USA). In a two-tailed Wald test, *p* < 0.05 was considered significant. OS and cancer-specific survival (CSS) were estimated using the Kaplan–Meier method, and differences among patients with non-COPD, COPD, and hospitalization for COPDAE were determined using the stratified log-rank test to compare survival curves (stratified according to matched sets) [28].

## *3.* Results

### 3.1. PSM and Study Cohort

PSM yielded a cohort of 708 patients with stage IIA–IVB MIBUC undergoing CCRT for bladder preservation (472 and 232 in Groups 1 and 2, respectively); their characteristics are summarized in Table 1. Age, sex, diabetes, hyperlipidemia, hypertension, AMI, cardiovascular diseases, ischemic stroke, kidney/bladder stones, CCI scores, AJCC clinical tumor stage, AJCC clinical nodal stage, surgical consolidation after CCRT, bladder preservation rate, cisplatin-based regimen dosage, and radiotherapy dosage were similar between the two groups due to PSM. All-cause death and hospitalization for COPDAE within 1 year before the index date were endpoints in our study and not matched between the two groups (Table 1).

### 3.2. All-Cause Mortality, COPD Death, and Bladder Cancer Death

Table 1 presents that after PSM, not only COPD death but also the all-cause death and bladder cancer death were significantly higher in Group 2 than in Group 1 (*p* < 0.001). Multivariate Cox regression analysis indicated that COPD and ≥1 or ≥2 hospitalizations for COPDAE within 1 year before CCRT for bladder preservation were associated with poor OS (Table 2). No significant differences were observed in age, sex, diabetes, hyperlipidemia, hypertension, AMI, cardiovascular diseases, ischemic stroke, kidney/bladder stones, CCI scores, AJCC clinical tumor stage, AJCC clinical nodal stage, surgical consolidation after CCRT, bladder preservation rate, cisplatin-based regimen dosage, and radiotherapy dosage (Table 2) because a well-matched head-to-head PSM design was used without residual imbalance [26,27]. The adjusted hazard ratio (aHR; 95% confidence interval (CI)) of all-cause mortality for Group 2 compared with Group 1 was 1.89 (1.12–3.18, *p* = 0.017). The aHRs (95% CIs) of all-cause mortality for ≥1 or ≥2 hospitalizations for COPDAE within 1 year before CCRT for bladder preservation were 3.26 (1.95–5.46; *p* < 0.0001) and 6.33 (3.55–11.28, *p* < 0.0001) compared with non-COPDAE patients with MIBUC undergoing CCRT for bladder preservation. In Supplemental Table S3, multivariable analysis shows COPDAE was still an independent significant prognostic factor of mortality compared with COPD without AE. The aHRs (95% CIs) of all-cause mortality for ≥1 or ≥2 hospitalizations for COPDAE within 1 year before CCRT for bladder preservation were 2.77 (1.65–4.64; *p* < 0.0001) and 5.38 (2.84–9.18, *p* < 0.0001) compared with COPD patients without AE with MIBUC undergoing CCRT for bladder preservation.

### 3.3. Kaplan–Meier OS among Non-COPD, COPD, and Hospitalization for COPDAE

**Figure 1:**
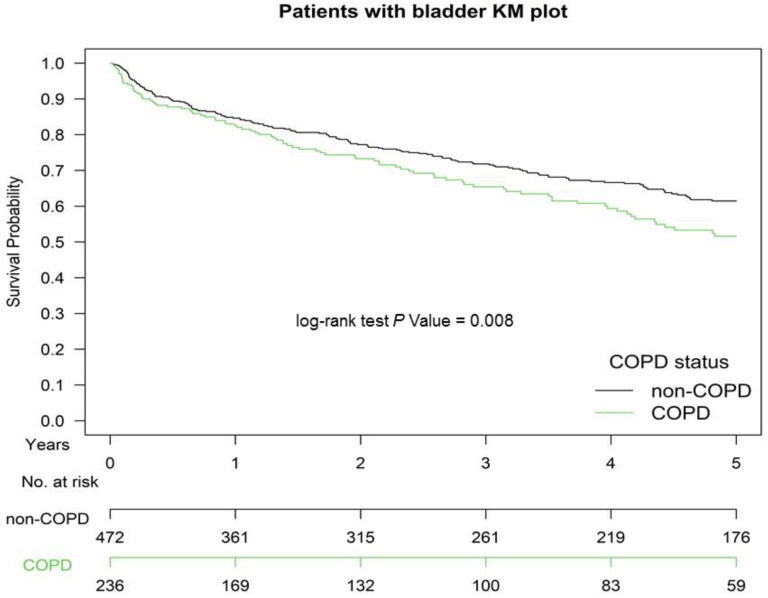
Kaplan–Meier (KM) survival curves of propensity score-matched patients with muscle-invasive urothelial carcinoma of the bladder with and without current-smoking-related COPD before definitive CCRT for bladder preservation, COPD, chronic obstructive pulmonary disease; CCRT, concurrent chemoradiotherapy.

**Figure 2:**
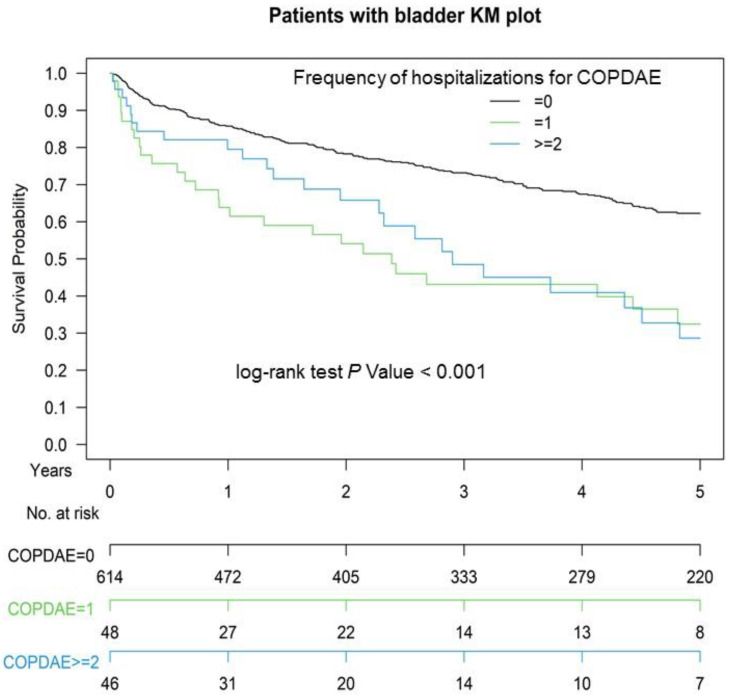
Kaplan–Meier (KM) survival curves of propensity score-matched patients with muscle-invasive urothelial carcinoma of the bladder with hospitalization frequency for COPDAE within 1 year before definitive CCRT for bladder preservation, COPDAE, chronic obstructive pulmonary disease with acute exacerbation; CCRT, concurrent chemoradiotherapy.

Figure 1 presents the Kaplan–Meier OS curves for the two groups. The OS of Group 2 was significantly inferior to that of Group 1 (*p* = 0.008). The OS in patients with ≥1 or ≥2 hospitalizations for COPDAE within 1 year before CCRT for bladder preservation was significantly inferior to that of patients with 0 hospitalizations for COPDAE (*p* < 0.001; Figure 2). The CSS of Group 2 was significantly inferior to that of Group 1 (*p* < 0.001; Supplemental Figure S1).

## 4. Discussion

Cigarette smoking is an etiologic factor for bladder cancer, and many patients have one or more tobacco-related comorbidities, which increase the risk of perioperative mortality [9,29,30]. Additionally, patients who smoke during bladder cancer treatment may experience worsened clinical outcomes, such as increased risk of recurrent disease, decreased response to chemotherapy, and increased mortality rates [9]. However, no study has estimated the association of smoking with comorbidities such as COPD and COPD severity (hospitalization for COPDAE was considered a severe COPD status and named as Global Initiative for Chronic Obstructive Lung Disease [GOLD] C–D stages [31]) for all-cause death in patients with MIBUC receiving CCRT for bladder preservation instead of surgery. Our study is the first to evaluate the prognosis of COPD and COPDAE in patients with bladder cancer receiving definitive CCRT. Our findings could be a valuable indicator of OS in patients with MIBUC receiving CCRT for bladder preservation. Prevention of COPD progression to COPDAE can be an essential health policy for increasing the OS in patients with MIBUC receiving CCRT for bladder preservation (Supplemental Table S3).

TURBT followed by CCRT with a cisplatin-based regimen has been the main treatment for bladder preservation in patients with MIBUC for >20 years [8,32,33,34]. TMT is a suboptimal treatment and should be offered as an alternative to selected, well-informed, and compliant patients, especially for whom radical cystectomy is not an option or not acceptable. In a systemic review, the five-year CSS and OS rates of TMT range from 50% to 82% and from 36% to 74%, respectively, with salvage cystectomy rates of 25–30% compatible with ours (five-year OS rate was 63.1% and 51.4% for non-COPD and COPD groups, respectively; Figure 1) [35]. Therefore, our standard TMT with reasonable survival rate in our study is compatible with that of other studies [35]. However, both preclinical and clinical studies have reported that cigarette smoking causes resistance to cisplatin [9,10,36,37,38]. In addition, smoking results in worse survival because of an increased recurrence rate after bladder cancer treatment [9,39,40]. The potential mechanism of smoking-induced resistance to cisplatin is changes in the expression of drug influx and efflux transporters, decreased uptake, inactivation by nucleophilic compounds, or accelerated DNA repair in smoking-exposed urothelial cells [37,41,42]. Moreover, COPD has been associated with poor survival in lung and extrapulmonary cancer treatments [43,44,45,46]. Patients with cancer having COPD have worse survival than those without COPD [43,44,45,46,47] because COPD increases C-reactive protein levels, a biomarker of systemic inflammation, which is associated with an increased risk of cancer mortality, including for extrapulmonary cancers [47]. Similarly, in the largest meta-analysis of its kind, Danesh and colleagues indicated that plasma fibrinogen, another nonspecific marker of systemic inflammation, is associated with both pulmonary and extrapulmonary cancers in smokers and never smokers [48]. Therefore, a reasonable assumption is that current-smoking-related COPD and COPD severity such as hospitalization for COPDAE before CCRT might be associated with worse survival in patients undergoing CCRT for MIBUC compared with those who never smoked and did not have COPD. In our study, we estimated COPD death and bladder cancer death from the Cause of Death database between the two groups. Bladder cancer death was still higher in Group 2 than in Group 1. Thus, the survival benefits of CCRT for bladder preservation in Group 2 were inferior to those in Group 1. In our study, current-smoking-related COPD was an independent prognostic factor for poor OS in patients with MIBUC receiving CCRT for bladder preservation after head-to-head PSM. Preexisting COPD within 1 year before CCRT was a useful indicator of prognostic factor for OS, which could be used as a reference for shared decision-making between physicians and patients in the future. In addition, high COPD severity, such as hospitalization for COPDAE (GOLD C and D stages) before CCRT, was associated with worsened survival in patients with MIBUC receiving CCRT for bladder preservation (Table 2, Figure 2, and Supplemental Table S3). Our findings imply that the prevention of COPD progression to COPDAE before CCRT would be crucial for increasing OS in patients with MIBUC receiving CCRT for bladder preservation (Supplemental Table S3).

In Table 1, all potential covariates associated with OS in propensity score-matched patients with COPD or bladder cancer are considered. The following covariates between the case and control groups were homogeneous (Table 1): age, sex, diabetes, hyperlipidemia, hypertension, AMI, cardiovascular diseases, ischemic stroke, kidney/bladder stones, CCI scores, AJCC clinical tumor stage, AJCC clinical nodal stage, surgical consolidation after CCRT, bladder preservation rate, cisplatin-based regimen dosage, and radiotherapy dosage. These covariates are prognostic factors for OS in patients with COPD or bladder cancer. COPD is the result of a complex interplay between clinical and molecular (i.e., genetic) risk factors [49]. Many COPD-related comorbidities and smoking increase COPD severity and mortality [50,51,52,53,54]. These comorbidities, such as diabetes, hyperlipidemia, hypertension, AMI, cardiovascular diseases, and ischemic stroke, have been considered for PSM [52,53,54]. Moreover, age, sex, kidney/bladder stones, CCI scores, AJCC clinical tumor stage, AJCC clinical nodal stage, surgical consolidation after CCRT, bladder preservation rate, cisplatin-based regimen dosage, and radiotherapy dosage might be prognostic factors for OS in patients with MIBUC receiving TURBT followed by CCRT for bladder preservation [55,56,57]; therefore, we considered them as covariates and included them in PSM. Most confounding factors were matched in the study. Therefore, COPD within 1 year before CCRT is associated with poor OS, and COPDAE within 1 year before CCRT is also associated with poor OS in patients having COPD without AE (Table 2, Figure 2, and Supplemental Table S3).

Although bladder preservation might be more effective for specific conditions such as smaller solitary tumors, negative nodes, no extensive or multifocal carcinoma in situ, no tumor-related hydronephrosis, and good pretreatment bladder function, physicians in Taiwan have also performed TMT based on NCCN guidelines for a small portion of patients who are lymph node-positive [6]. In our study, more than 60% of cN0 patients with MIBUC were undergoing TURBT followed by CCRT for bladder preservation (Table 1). In fact, there were patients with MIBUC who were lymph node-positive undergoing TURBT followed by CCRT for bladder preservation in Taiwan [58], which is compatible with the other studies [59,60]. Therefore, the small portion of patients who were lymph node-positive was a reasonable in the real world. Moreover, through PSM, the clinical N stages were balanced between case and control cohorts (Table 1), supporting our hypothesis that COPD before CCRT was an independent risk factor for mortality in these patients. 

All potential confounding factors were matched and had no residual imbalance without statistical significance in the covariates (Table 2) [26,27]. The independent prognostic factor for OS was preexisting COPD in patients with MIBUC (Table 2, Figure 1 and Figure 2). Hospitalization for COPDAE within 1 year before CCRT was an independent risk factor for mortality in these COPD patients (Supplemental Table S3). This valuable information will act as a reference for shared decision-making between physicians and patients regarding treatment for MIBUC. Moreover, preexisting COPD and COPDAE before CCRT for bladder preservation could be considered in future clinical trials to correct confounding factors. In addition, the prevention of preexisting COPD progression to COPDAE is crucial for patients receiving TURBT followed by CCRT as curative-intent treatments (Supplemental Table S3). Bladder cancer death was significantly higher in Group 2 than in Group 1 (Table 1 and Supplemental Figure S1). The findings indicate that the anticancer effect of CCRT among patients with MIBUC might be attenuated in Group 2 compared with Group 1 (Table 1 and Supplemental Figure S1). Only COPD death did not contribute to all-cause mortality in the COPD group because bladder cancer death was significantly higher in Group 2 than in Group 1.

Emerging evidence revealed that cigarette smoking might induce a mechanism of resistance to cisplatin-based chemotherapy in BC [10,12].

Cigarette smoking may induce resistance to cisplatin-based chemotherapy for bladder cancer [9,10]. Moreover, smoking during therapy for tobacco-related cancers is associated with decreased response rates to chemotherapy and radiotherapy [10,61]. For example, patients with head and neck cancer or lung cancer who continue to smoke during radiotherapy have lower response rates and survival rates than patients who do not smoke during radiotherapy [61,62]. Clinical studies have demonstrated that patients with lung cancer with severe or very severe COPD had a lower OS than did those without COPD or those with mild or moderate COPD [43,45]. However, these studies scarcely focus on the survival impact of COPD on patients with nonlung cancers. Taken together, these findings imply that both smoking and COPD result in poor OS and worse CSS in patients with cancers receiving cisplatin-based chemotherapy and radiotherapy as curative-intent treatments. CCRT for bladder preservation using cisplatin-based regimen might be influenced by current-smoking-related COPD [37,41,42,43,44,45,46,47]. Hospitalization for COPDAE within 1 year before CCRT is associated with COPD severity and is proportional to the smoking level. Thus, preexisting COPD and COPDAE may be valuable surrogates as prognostic factors for OS and CSS in patients with bladder cancer receiving CCRT (Figure 1 and Supplemental Figure S1). COPD and COPDAE, instead of smoking levels, are easily useful comorbidities as prognostic factors for OS and CSS in patients with MIBUC receiving CCRT for bladder preservation.

This is the first and largest cohort study to estimate the survival outcomes of current smokers with smoking-related COPD compared with nonsmokers without COPD having MIBUC and receiving TURBT followed by CCRT for bladder preservation based on the NCCN guidelines [6]. PSM led to comparable covariates between groups, and no selection bias was noted. No study has estimated the impact of preexisting COPD and hospitalization for COPDAE within 1 year before CCRT on patients with MIBUC undergoing bladder preservation. Furthermore, most confounding factors were controlled in our study. Our findings may serve as references for shared decision-making by physicians and patients who choose CCRT for bladder preservation. Moreover, prevention of COPD progression to COPDAE is crucial to increase OS in patients with MIBUC receiving CCRT for bladder preservation (Supplemental Table S3).

This study has some limitations. First, our cohort of patients with MIBUC was derived from an Asian population. However, no evidence has indicated differences between the oncologic outcomes of Asian and non-Asian patients with MIBUC undergoing TURBT followed by CCRT for bladder preservation. Second, the diagnoses of all comorbid conditions were based on *ICD-10-CM* codes. The Taiwan Cancer Registry Administration randomly reviews medical records and interviews patients to verify the accuracy of the diagnoses, and hospitals with outlier charges or practices are audited and heavily penalized if malpractice or discrepancies are identified. Nevertheless, to obtain crucial information on population specificity and disease occurrence, a large-scale randomized trial comparing carefully selected patients undergoing suitable treatments is essential. Third, selection bias and residual or unmeasured confounding are likely, as in all retrospective studies. Despite these limitations, a major strength of this study is the use of a nationwide population-based registry with detailed baseline and treatment information. Lifelong follow-up was possible through the linkage of the registry with the national Cause of Death database. Considering the magnitude and statistical significance of the observed effects in the current study, the limitations are unlikely to affect our conclusions.

## 5. Conclusions

Among patients with MIBUC undergoing TURBT followed by CCRT for bladder preservation, current smokers with smoking-related COPD had worse survival outcomes than did nonsmokers without COPD in terms of both bladder cancer death and all-cause mortality. Hospitalization for COPDAE within 1 year before CCRT was an independent risk factor for mortality in these COPD patients. The prevention of COPD progression to COPDAE was associated with an increase in OS in COPD patients who received bladder preservation through CCRT.

## Figures and Tables

**Table 1:** Characteristics of propensity score-matched patients with muscle-invasive urothelial carcinoma of the bladder with and without current-smoking-related COPD before definitive CCRT for bladder preservation.

	Never Smokers without COPD		Current Smokers with COPD		*p*
	N = 472	(100%)	N = 236	(100%)	
Age (mean ± SD)	(76.14 ± 8.22)		(76.22 ± 9.63)		0.844
Age (years)					1.000
≤65	58	12.29%	29	12.29%	
66–74	146	30.93%	73	30.93%	
75–85	178	37.71%	89	37.71%	
>85	90	19.07%	45	19.07%	
Sex					1.000
Female	116	24.58%	58	24.58%	
Male	356	75.42%	178	75.42%	
Diabetes					0.515
No	321	68.01%	154	65.25%	
Yes	151	31.99%	82	34.75%	
Hyperlipidemia					0.796
No	324	68.64%	165	69.92%	
Yes	148	31.36%	71	30.08%	
Hypertension					0.795
No	330	69.92%	162	68.64%	
Yes	142	30.08%	74	31.36%	
AMI					1.000
No	446	94.49%	223	94.49%	
Yes	26	5.51%	13	5.51%	
Cardiovascular diseases					0.453
No	398	84.32%	193	81.78%	
Yes	74	15.68%	43	18.22%	
Ischemic stroke					0.363
No	415	87.92%	201	85.17%	
Yes	57	12.08%	35	14.83%	
Kidney or bladder stones					0.202
No	349	73.94%	163	69.07%	
Yes	123	26.06%	73	30.93%	
CCI score					0.952
0	228	48.31%	116	49.15%	
≥1	244	51.69%	120	50.85%	
AJCC clinical tumor stage					1.000
cT2a	114	24.15%	57	24.15%	
cT2b	118	25.00%	59	25.00%	
cT3	166	35.17%	83	35.17%	
cT4	74	15.68%	37	15.68%	
AJCC clinical nodal stage					1.000
cN0	292	61.86%	146	61.86%	
cN1	136	28.81%	68	28.81%	
cN2	44	9.32%	22	9.32%	
Surgical consolidation after CCRT					1.000
No	354	75.00%	177	75.00%	
Yes	118	25.00%	59	25.00%	
Bladder preservation rate					1.000
No	164	34.75%	82	34.75%	
Yes	308	65.25%	154	65.25%	
Cisplatin-based regimen (cumulative total dose of cisplatin, mg/m^2^)					0.631
Median (Q1, Q3)	211.23	(206.43–276.21)	213.54	(210.12–281.52)	
Radiotherapy (total dose, Gy)					1.000
Median (Q1, Q3)	63.00	(61.20–64.80)	63.00	(61.20–64.80)	
Hospitalization frequency for COPDAE (within 1 year before CCRT)					<0.001
0	472	100.00%	142	60.17%	
1	0	0.00%	48	20.34%	
≥2	0	0.00%	46	19.49%	
Follow-up time Years (mean ± SD)	(5.71 ± 2.27)		(4.36 ± 2.19)		<0.001
COPD death					<0.001
Yes	0	0%	7	2.97%	
Bladder cancer death					<0.001
Yes	207	43.86%	133	56.36%	
All-cause death					<0.001
Yes	269	56.99%	177	75.00%	

SD, standard deviation; AJCC, American Joint Committee on Cancer; CCI, Charlson comorbidity index; COPD, chronic obstructive pulmonary disease; COPDAE, COPD with acute exacerbation; T, tumor; N, node; cT, clinical tumor stage; cN, clinical nodal stage; AMI, acute myocardial infarction; CCRT, concurrent chemoradiotherapy

**Table 2:** Cox proportional hazards analysis of all-cause mortality for patients with muscle-invasive urothelial carcinoma of the bladder with and without current-smoking-related COPD before definitive CCRT.

	Crude HR (95% CI)		Adjusted HR ^*^ (95% CI)		*p*
COPD status (ref. non-COPD)					
COPD	2.18	(1.30–3.64)	1.89	(1.12–3.18)	0.017
Hospitalization frequency for COPDAE before CCRT (ref. = 0)					
1	3.64	(2.20–6.03)	3.26	(1.95–5.46)	<0.001
≥2	7.93	(4.55–13.79)	6.33	(3.55–11.28)	<0.001
Sex (ref. Female)					
Male	0.85	(0.67–1.09)	1.02	(0.78–1.34)	0.865
Age (years; ref. ≤65 years)					
66–74	1.24	(0.97–1.57)	1.02	(0.77–1.33)	0.910
75–85	2.45	(0.76–3.59)	1.26	(0.80–1.98)	0.313
>85	2.70	(0.70–4.28)	1.28	(0.76–2.15)	0.355
CCI score (ref. = 0)					
≥1	1.58	(0.86–3.23)	1.26	(0.87–2.62)	0.821
Diabetes (ref.: No)					
Yes	1.25	(0.90–1.73)	1.19	(0.87–1.61)	0.317
Hyperlipidemia (ref.: No)					
Yes	1.19	(0.93–1.54)	1.07	(0.81–1.42)	0.630
Hypertension (ref.: No)					
Yes	1.06	(0.83–1.36)	1.09	(0.84–1.43)	0.520
AMI (ref.: No)					
Yes	1.28	(0.96–1.73)	1.18	(0.92–1.44)	0.740
Cardiovascular diseases (ref.: No)					
Yes	1.06	(0.79–1.42)	0.96	(0.85–1.28)	0.441
Ischemic stroke (ref.: No)					
Yes	1.26	(0.81–1.96)	1.21	(0.88–1.33)	0.463
Kidney or bladder stones (ref.: No)					
Yes	1.11	(0.86–1.43)	0.94	(0.72–1.22)	0.643
AJCC clinical tumor stages (ref. cT2a)					
cT2b	1.92	(0.72–5.16)	1.31	(0.47–3.63)	0.602
cT3	1.96	(0.92–4.17)	1.39	(0.64–3.05)	0.405
cT4	2.12	(0.98–4.59)	1.13	(0.50–2.53)	0.767
AJCC clinical nodal stages (ref. cN0)					
cN1	1.11	(0.86–1.43)	0.93	(0.72–1.21)	0.597
cN2	1.06	(0.83–1.36)	1.08	(0.83–1.41)	0.568
Surgical consolidation after CCRT (ref.: No)					
Yes	1.06	(0.64–1.14)	1.07	(0.60–1.11)	0.191
Bladder preservation (ref.: No)					
Yes	0.86	(0.68–1.09)	0.92	(0.79–1.03)	0.255

HR, hazard ratio; CI, confidence interval; AJCC, American Joint Committee on Cancer; CCI, Charlson comorbidity index; COPD, chronic obstructive pulmonary disease; COPDAE, COPD with acute exacerbation; T, tumor; N, node; cT, clinical tumor stage; cN, clinical nodal stage; AMI, acute myocardial infarction; CCRT, concurrent chemoradiotherapy. * All covariates mentioned in Table 2 were adjusted.

## Data Availability

Restrictions apply to the availability of these data. Data was obtained from Taiwan Ministry of Health and Welfare and are available from Szu-Yuan Wu with the permission of Institutional Review Board of Tzu-Chi Medical Foundation (IRB109-015-B).

## Supplementary Materials

The following are available online at https://www.mdpi.com/article/10.3390/jpm11100958/s1, Figure S1: Kaplan–Meier (KM) cancer-specific survival curves of propensity score–matched patients with muscle-invasive urothelial carcinoma of the bladder with and without current-smoking-related COPD before definitive CCRT for bladder preservation, Table S1: Characteristics of Patients With Muscle-Invasive Urothelial Carcinoma of the Bladder With and Without Current-Smoking-Related COPD Before Definitive CCRT for Bladder Preservation before Propensity Score–Matching, Table S2: Characteristics of COPD or COPDAE Patients With Muscle-Invasive Urothelial Carcinoma of the Bladder With and Without Current-Smoking-Related COPD Before Definitive CCRT for Bladder Preservation, Table S3: Cox Proportional Hazards Analysis of All-Cause Mortality for COPD or COPDAE Patients With Muscle-Invasive Urothelial Carcinoma Before Definitive CCRT.

## Abbreviations

COPD, chronic obstructive pulmonary disease; COPDAE, COPD with acute exacerbation; aHR, adjusted hazard ratio; CI, confidence interval; GOLD, Global Initiative for Chronic Obstructive Lung Disease; MIBUC, muscle-invasive bladder urothelial carcinoma; TURBT, transurethral resection of bladder tumor; CCRT, concurrent chemoradiotherapy; TMT, trimodality bladder preservation treatment; AJCC, American Joint Committee on Cancer; CCI, Charlson comorbidity index; T, tumor; N, nodal; cT, clinical tumor stage; cN, clinical nodal stage; AMI, acute myocardial infarction; NCCN, National Comprehensive Cancer Network; OS, overall survival; CSS, cancer-specific survival; PSM, propensity score matching; *ICD-10-CM*, *International Classification of Diseases, 10th Revision, Clinical Modification.*
