# Supplementary material for: Survival Impact of Current-Smoking-Related COPD or COPD with Acute Exacerbation on Bladder Preservation through Concurrent Chemoradiotherapy for Muscle-Invasive Bladder Urothelial Carcinoma
Source: J Pers Med. 2021 Sep 26;11(10):958. doi: 10.3390/jpm11100958 (PMC8539205; doi:10.3390/jpm11100958)
Supplement: Supplementary file 1 [file jpm-11-00958-s001.zip › jpm-1370650-supplementary.pdf]

Journal of Personalized Medicine

MDPI

Supplemental Table 1. Characteristics of Patients With Muscle-Invasive Urothelial Carcinoma of the Bladder With and Without Current-Smoking-Related COPD Before Definitive CCRT for Bladder Preservation before Propensity Score–Matching

	Never Smokers Without COPD N = 1145 (100%)		Current Smokers With COPD N = 236 (100%)		*P*
Age (mean ± SD)	(67.99 ± 12.88)		(76.22 ± 9.63)		< 0.001
Age (years)					<0.001
≤65	374	32.66%	29	12.29%	
66–74	334	29.17%	73	30.93%	
75–85	266	23.23%	89	37.71%	
>85	171	14.93%	45	19.07%	
Sex					0.079
Female	350	30.57%	58	24.58%	
Male	795	69.43%	178	75.42%	
Diabetes					<0.001
No	886	77.38%	154	65.25%	
Yes	259	22.62%	82	34.75%	
Hyperlipidemia					0.025
No	882	77.03%	165	69.92%	
Yes	263	22.97%	71	30.08%	
Hypertension					<0.001
No	904	78.95%	162	68.64%	
	241	21.05%	74	31.36%	

Yes					0.078
No	1,111	97.03%	223	94.49%	
Yes	34	2.97%	13	5.51%	
Cardiovascular diseases					<0.001
No	1,051	91.79%	193	81.78%	
Yes	94	8.21%	43	18.22%	
Ischemic stroke					<0.001
No	1,072	93.62%	201	85.17%	
Yes	73	6.38%	35	14.83%	
Kidney or bladder stones					0.367
No	827	72.23%	163	69.07%	
Yes	318	27.77%	73	30.93%	
CCI score					<0.001
0	890	77.73%	116	49.15%	
≥1	255	22.27%	120	50.85%	
AJCC clinical tumor stage					<0.001
cT2a	386	33.71%	57	24.15%	
cT2b	388	33.89%	59	25.00%	
cT3	214	18.69%	83	35.17%	
cT4	157	13.71%	37	15.68%	
AJCC clinical nodal stage					<0.001
cN0	820	71.62%	146	61.86%	
cN1	271	23.67%	68	28.81%	

cN2	54	4.71%	22	9.32%	
Surgical consolidation after CCRT					0.042
No	914	79.83%	177	75.00%	
Yes	231	20.17%	59	25.00%	
Bladder preservation rate					0.031
No	343	29.96%	82	34.75%	
Yes	802	70.04%	154	65.25%	
Cisplatin-based regimen (cumulative total dose of cisplatin, mg/m^2)					0.519
Median (Q1, Q3)	223.81	(202.17–279.11)	213.54	(210.12–281.52)	
Radiotherapy (total dose, Gy)					1.000
Median (Q1, Q3)	63.00	(61.20–64.80)	63.00	(61.20–64.80)	
Hospitalization frequency for COPDAE (within 1 year before CCRT)					<0.001
0	1145	100.00%	142	60.17%	
1	0	0.00%	48	20.34%	
≥2	0	0.00%	46	19.49%	
Follow-up time Years (mean ± SD)	(5.90 ± 2.43)		(4.36 ± 2.19)		<0.001
COPD death					<0.001
Yes	0	0%	7	2.97%	
Bladder cancer death					<0.001
Yes	482	42.10%	133	56.36%	
All-cause death					<0.001
Yes	643	56.16%	177	75.00%	

SD, standard deviation; AJCC, American Joint Committee on Cancer; CCI, Charlson comorbidity index; COPD, chronic obstructive pulmonary disease; COPDAE, COPD with acute exacerbation; T, tumor; N, node; cT, clinical tumor stage; cN, clinical nodal stage; AMI, acute myocardial infarction; CCRT, concurrent chemoradiotherapy

Supplemental Table 2. Characteristics of COPD or COPDAE Patients With Muscle-Invasive Urothelial Carcinoma of the Bladder With and Without Current-Smoking-Related COPD Before Definitive CCRT for Bladder Preservation

	COPDAE		COPD		*P*
	N = 94 (100%)		N = 142 (100%)		
Age (mean ± SD)	(77.52 ± 8.91)		(75.08 ± 10.11)		< 0.001
Age (years)					<0.001
≤65	9	9.57%	20	14.08%	
66–74	24	25.53%	49	33.11%	
75–85	38	40.43%	51	35.92%	
>85	23	24.47%	22	14.86%	
Sex					<0.001
Female	15	15.96%	43	30.28%	
Male	79	84.04%	99	69.72%	
Diabetes					<0.001
No	56	59.57%	98	69.01%	
Yes	38	40.43%	44	30.99%	
Hyperlipidemia					<0.001
No	57	60.64%	108	76.06%	

Yes	37	39.36%	34	23.94%	
Hypertension					<0.001
No	61	64.89%	101	71.13%	
Yes	33	35.11%	41	28.87%	
AMI					0.041
No	87	92.55%	136	95.77%	
Yes	7	7.45%	6	4.23%	
Cardiovascular diseases					<0.001
No	74	78.72%	119	83.80%	
Yes	20	21.28%	23	16.20%	
Ischemic stroke					0.032
No	76	80.85%	125	88.03%	
Yes	18	19.15%	17	11.97%	
Kidney or bladder stones					0.285
No	66	70.21%	97	68.31%	
Yes	28	29.79%	45	31.69%	
CCI score					<0.001
0	37	39.36%	79	55.63%	
≥1	57	60.64%	63	44.37%	
AJCC clinical tumor stage					<0.001
cT2a	13	13.83%	44	30.99%	
cT2b	28	29.79%	31	21.83%	
cT3	38	40.43%	45	31.69%	

cT4	15	15.96%	22	15.49%	
AJCC clinical nodal stage					<0.001
cN0	47	50.00%	99	69.72%	
cN1	33	35.11%	35	24.65%	
cN2	14	14.89%	8	5.63%	
Surgical consolidation after CCRT					<0.001
No	65	69.15%	112	78.87%	
Yes	29	30.85%	30	21.13%	
Bladder preservation rate					<0.001
No	40	42.55%	42	29.58%	
Yes	54	57.45%	100	70.42%	
Cisplatin-based regimen (cumulative total dose of cisplatin, mg/m^2)					0.382
Median (Q1, Q3)	209.81	(200.98–268.32)	215.74	(208.78–281.4)	
Radiotherapy (total dose, Gy)					1.000
Median (Q1, Q3)	63.00	(61.20–64.80)	63.00	(61.20–64.80)	
Follow-up time Years (mean ± SD)	(4.01 ± 2.10)		(4.58 ± 2.29)		<0.001
COPD death					<0.001
Yes	7	7.45%	0	0.00%	
Bladder cancer death					<0.001
Yes	58	61.70%	75	52.81%	
All-cause death					<0.001
Yes	79	84.04%	98	69.01%	

SD, standard deviation; AJCC, American Joint Committee on Cancer; CCI, Charlson comorbidity index; COPD, chronic obstructive pulmonary disease; COPDAE, COPD with acute exacerbation; T, tumor; N, node; cT, clinical tumor stage; cN, clinical nodal stage; AMI, acute myocardial infarction; CCRT, concurrent chemoradiotherapy

Supplemental Table 3. Cox Proportional Hazards Analysis of All-Cause Mortality for COPD or COPDAE Patients With Muscle-Invasive Urothelial Carcinoma Before Definitive CCRT

	Crude HR (95% CI)		Adjusted HR (95% CI)		*P*
Hospitalization frequency for COPDAE before CCRT (ref. = 0)					
1	3.09	(1.87–5.13)	2.77	(1.65–4.64)	<0.001
≥2	6.74	(3.86–9.05)	5.38	(2.84–9.18)	<0.001
Sex (ref. Female)					
Male	1.15	(1.07–1.29)	1.12	(1.03–1.24)	0.025
Age (years; ref. ≤65 years)					
66–74	1.03	(0.88–1.32)	1.06	(0.89–1.29)	0.668
75–85	1.35	(1.03–1.68)	1.31	(1.10–1.52)	0.019
> 85	1.92	(1.17–2.01)	1.82	(1.19–1.92)	0.005
CCI score (ref. = 0)					
≥1	1.42	(1.06–2.19)	1.40	(1.07–2.05)	0.008
Diabetes (ref.: No)					
Yes	1.02	(0.90–1.42)	1.03	(0.79–1.32)	0.132

Hyperlipidemia (ref.: No)					
Yes	1.22	(0.71–1.91)	1.21	(0.80–1.53)	0.518
Hypertension (ref.: No)					
Yes	1.11	(0.76–1.41)	1.07	(0.74–1.40)	0.462
AMI (ref.: No)					
Yes	1.14	(0.81–1.52)	1.11	(0.80–1.51)	0.616
Cardiovascular diseases (ref.: No)					
Yes	1.02	(1.00–1.50)	1.01	(0.95–1.48)	0.113
Ischemic stroke (ref.: No)					
Yes	1.20	(0.69–1.88)	1.19	(0.68–1.83)	0.371
Kidney or bladder stones (ref.: No)					
Yes	1.08	(0.75–1.58)	1.04	(0.71–1.39)	0.418
AJCC clinical tumor stages (ref. cT2a)					
cT2b	1.31	(0.92–4.01)	1.28	(0.97–3.48)	0.096
cT3	1.58	(1.02–4.01)	1.49	(1.12–3.83)	0.006
cT4	2.26	(1.08–4.27)	2.12	(1.05–3.99)	0.007
AJCC clinical nodal stages (ref. cN0)					
cN1	1.29	(1.16–3.10)	1.23	(1.12–2.71)	0.002
cN2	1.48	(1.81–4.31)	1.31	(1.21–4.17)	0.008
Surgical consolidation after CCRT (ref.: No)					
Yes	1.12	(0.71–1.64)	1.08	(0.70–1.59)	0.304
Bladder preservation (ref.: No)					

Yes	0.83	(0.48–1.13)	0.82	(0.59–1.09)	0.324

HR, hazard ratio; CI, confidence interval; AJCC, American Joint Committee on Cancer; CCI, Charlson comorbidity index; COPD, chronic obstructive pulmonary disease; COPDAE, COPD with acute exacerbation; T, tumor; N, node; cT, clinical tumor stage; cN, clinical nodal stage; AMI, acute myocardial infarction; CCRT, concurrent chemoradiotherapy

*All covariates mentioned in Table 2 were adjusted.

Supplemental Figure 1. Kaplan–Meier (KM) cancer-specific survival curves of propensity score–matched patients with muscle-invasive urothelial carcinoma of the bladder with and without current-smoking-related COPD before definitive CCRT for bladder preservation

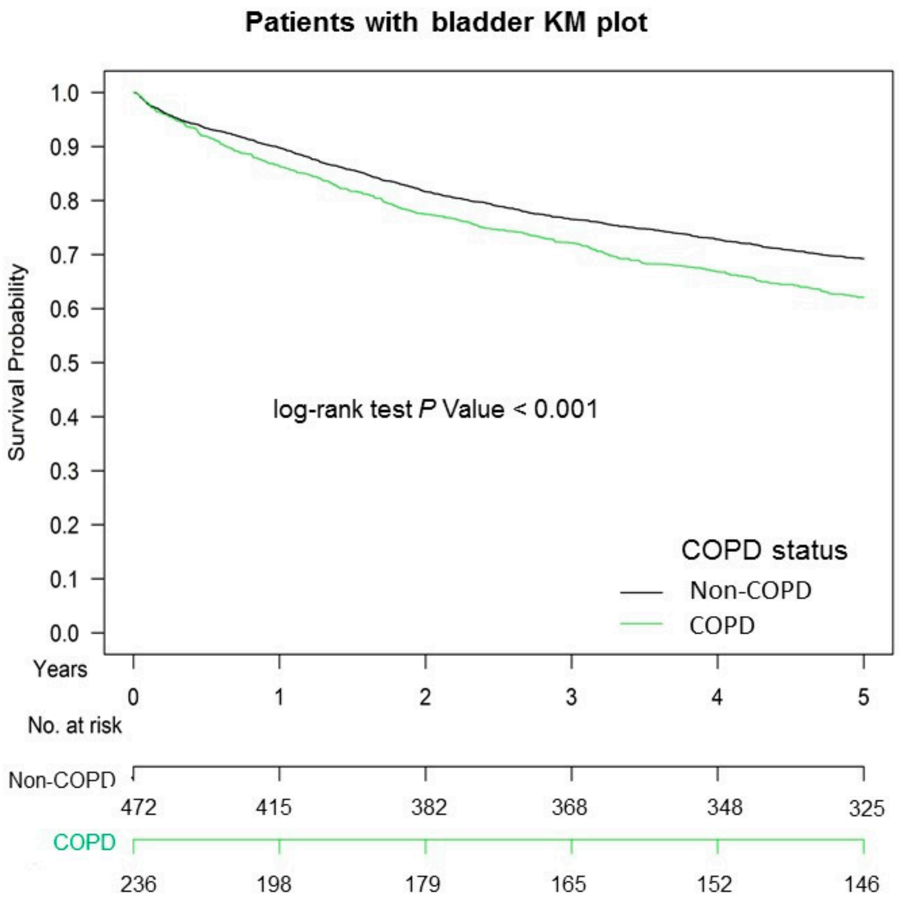
Patients with bladder KM plot
Survival Probability
1.0
0.9
0.8
0.7
0.6
0.5
0.4
0.3
0.2
0.1
0.0
log-rank test P Value < 0.001
COPD status
Non-COPD
COPD
Years
0
1
2
3
4
5
No. at risk
Non-COPD
472
415
382
368
348
325
COPD
236
198
179
165
152
146
